# Small cell and non small cell lung cancer form metastasis on cellular 4D lung model

**DOI:** 10.1186/s12885-018-4358-x

**Published:** 2018-04-18

**Authors:** Dhruva K. Mishra, Ross A. Miller, Kristi A. Pence, Min P. Kim

**Affiliations:** 1 Department of Surgery, Houston Methodist Hospital Research Institute, Houston, TX USA; 2 Department of Pathology and Genomic Medicine, Houston Methodist Hospital, Houston, TX USA; 3 Division of Thoracic Surgery, Department of Surgery, Weill Cornell Medical College, Houston Methodist Hospital, 6550 Fannin Street, Suite 1661, Houston, TX 77030 USA

**Keywords:** 4D cellular model, Lung Cancer, Breast cancer

## Abstract

**Background:**

Metastasis is the main cause of death for lung cancer patients. The ex vivo 4D acellular lung model has been shown to mimic this metastatic process. However, the main concern is the model’s lack of cellular components of the tumor’s microenvironment. In this study, we aim to determine if the intact lung microenvironment will still allow lung cancer metastasis to form.

**Methods:**

We harvested a heart-lung block from a rat and placed it in a bioreactor after cannulating the pulmonary artery, trachea and tying the right main bronchus for 10–15 days without any tumor cells as a control group or with NSCLC (A549, H1299 or H460), SCLC (H69, H446 or SHP77) or breast cancer cell lines (MCF7 or MDAMB231) through the trachea. We performed lobectomy, H&E staining and IHC for human mitochondria to determine the primary tumor’s growth and formation of metastatic lesions. In addition, we isolated circulating tumor cells (CTC) from the model seeded with GFP tagged cells.

**Results:**

In the control group, no gross tumor nodules were found, H&E staining showed hyperplastic cells and IHC showed no staining for human mitochondria. All of the models seeded with cancer cell lines formed gross primary tumor nodules that had microscopic characteristics of human cancer cells on H&E staining with IHC showing staining for human mitochondria. CTC were isolated for those cells labeled with GFP and they were viable in culture. Finally, all cell lines formed metastatic lesions with cells stained for human mitochondria.

**Conclusion:**

The cellular ex vivo 4D model shows that human cancer cells can form a primary tumor, CTC and metastatic lesions in an intact cellular environment. This study suggests that the natural matrix scaffold is the only necessary component to drive metastatic progression and that cellular components play a role in modulating tumor progression.

**Electronic supplementary material:**

The online version of this article (10.1186/s12885-018-4358-x) contains supplementary material, which is available to authorized users.

## Background

Stage IV, the point in tumor progression in which cancer spreads beyond the primary site and regional lymph nodes and is found in other organs, is the cancer stage that most often leads to patient mortality [1]. The tumor’s microenvironment plays a critical role in tumor growth and the development of metastasis where the interaction between tumor cells and the associated stroma and cellular components modulates the tumor’s progression and patient prognosis. Recently, the acellular 4D lung model has successfully mimicked the development of metastasis [2]. It is named the 4D model because of its perfusion of tumor nodules that allows it to change over time and grow in the 3D space. Findings from the 4D model suggest that the only component of tumor microenvironment that is important to show tumor progression is an intact natural matrix [2].

The acellular 4D lung model is created by removing all of the cells from a rat heart and lung block [3, 4]. This natural lung matrix maintains its three-dimensional architecture, including perfusable vascular beds and preserved airways. The matrix is composed of collagen, proteoglycans, and elastic fibers that preserve the architecture of airways and capillaries. A unique feature of the matrix is that this composition is preserved among species in the distal airways [5]. Furthermore, the basement membranes of the alveolar septa are preserved after decellularization in this model [3]. The acellular 4D lung model shows that when tumor cells are placed into the trachea, they form perusable nodules in the lung matrix [6]. Moreover, the model allows tumor cells to secrete proteins that are more similar those found in lung cancer patients than the same tumor cells grown on a petri dish [7]. The acellular 4D lung model mimics metastasis, with the placement of all tumor cells in the left lung lobes and perfusion of the model in the bioreactor through the pulmonary artery. In order for the tumor cells to enter the right lung, the cells would need to leave the epithelial space in the left side, enter the vasculature, and enter the other epithelial space on the right side. Over time, this process occurred as metastatic lesions formed in the right lung and grew over time in the 4D model [2]. There are significant differences in the spatial organization of the tumor cells where the primary tumor grew in a pattern along the airway and the metastatic lesion formed in a distribution that is consistent with cancer distributed along the vasculature. The model’s unique vascular channel allowed dead cells as well as live circulating tumor cells (CTC) to enter the vasculature. The CTC showed differences in behavior and gene expression compared to those cells initially placed in the model. The CTC took longer to attach to the petri dish than the parental cells placed in the model and they stayed alive in supernatant with decreased expression of integrin beta 4 (ITGB4) [8]. In addition, CTCs were resistant to chemotherapy [9]. There is no difference in the number of live CTC from the 4D model when they are placed in the petri dish, with or without Cisplatin, while the same dose for the parental cells (2D) placed in the model showed a significant reduction of live cells [9]. Previous studies also show that the CTC form metastatic lesions in the 4D model [2].

A major drawback of this acellular 4D model has been the lack of cellular components that are found in a patient’s tumor microenvironment. These studies suggest that the natural matrix architecture is the only component necessary for complex perfusable nodules to form, the creation of CTC, and ultimately metastatic lesion formation. However, one could argue that this phenomenon is simply due to the artificial creation of an acellular environment. Thus, in this study, we show that the ex vivo 4D lung model can mimic the metastatic process in a normal cellular environment. We postulate that non-small cell and small cell lung cancer cell lines as well as breast cancer cell lines will grow in the model and form a primary tumor, CTC, and metastatic lesions.

## Methods

All of the animal experiments were carried out in accordance with all applicable laws, regulations, guidelines, and policies governing the use of laboratory animal in research. The Institutional Animal Care and Use Committee (IUCAC) at the Houston Methodist Research Institute approved the protocols for animal experiments.

### Rat lung isolation

We harvested the lung–heart block from 4 to 6 week old male Sprague-Dawley rats as previously described [6]. Briefly, we euthanized Sprague-Dawley rats with ketamine (100 mg/mL) and xylazine (10 mg/mL) and performed bilateral thoracotomy to open the thoracic cavity. We injected 2 mL of heparin (1000 units/mL, Sagent Pharmaceuticals, IL, USA) into the right ventricle of heart, removed the rib cage and injected 20 mL of heparinized Phosphate Buffered Saline (12.5 Units/mL) in the right ventricle after placing an 18-gauge needle (McMaster Carr, USA) in the left ventricle as a vent. The superior vena cava and inferior vena cava were cut and the lungs were flushed again with 20 mL of heparinized PBS. Next, we divided the trachea at the level of the thyroid, the branches of the aorta at the arch, and the descending aorta at the level of the hemiazygos vein. The heart-lung block was then separated from the esophagus and the rest of the rat body. We performed ventriculotomy to expose the right and left ventricles and placed a custom-made prefilled 18-gauge stainless steel needle (McMaster Carr, USA) through the right ventricle into the main pulmonary artery. This was secured with a 2–0 silk tie (Ethicon, San Angelo, TX, USA). We also placed a female luer bulkhead (Cole-Parmer, IL, USA) in the left ventricle and secured it with a 2–0 silk tie. We flushed the pulmonary artery cannula with heparinized PBS and placed it in a container containing heparinized PBS.

### Cell culture

Human cancer cell lines A549, H1299, H460, H69, H446, SHP-77, MCF7, and MDAMB231 were obtained from ATCC (Manassas, VA, USA). These cell lines were grown in BD T175 cell culture flasks in RPMI 1640 medium (Hyclone, UT, USA) supplemented with 10% fetal bovine serum (Gibco, USA) and antibiotics (100 IU/mL penicillin, 100 mg/mL streptomycin, and 0.25 mg/mL amphotericin; Gibco, USA) at 37°C in 5% CO2. Once the cells were 85% confluent, they were washed with PBS and subjected to trypsinization using 0.25% trypsin (Gibco, USA) to collect the cells from flasks. The cells were washed with medium and finally suspended in 50 mL of complete culture medium. Approximately 15 million cells were used to seed the lung matrix.

### Bioreactor for the cellular 4D lung

**Fig. 1:**
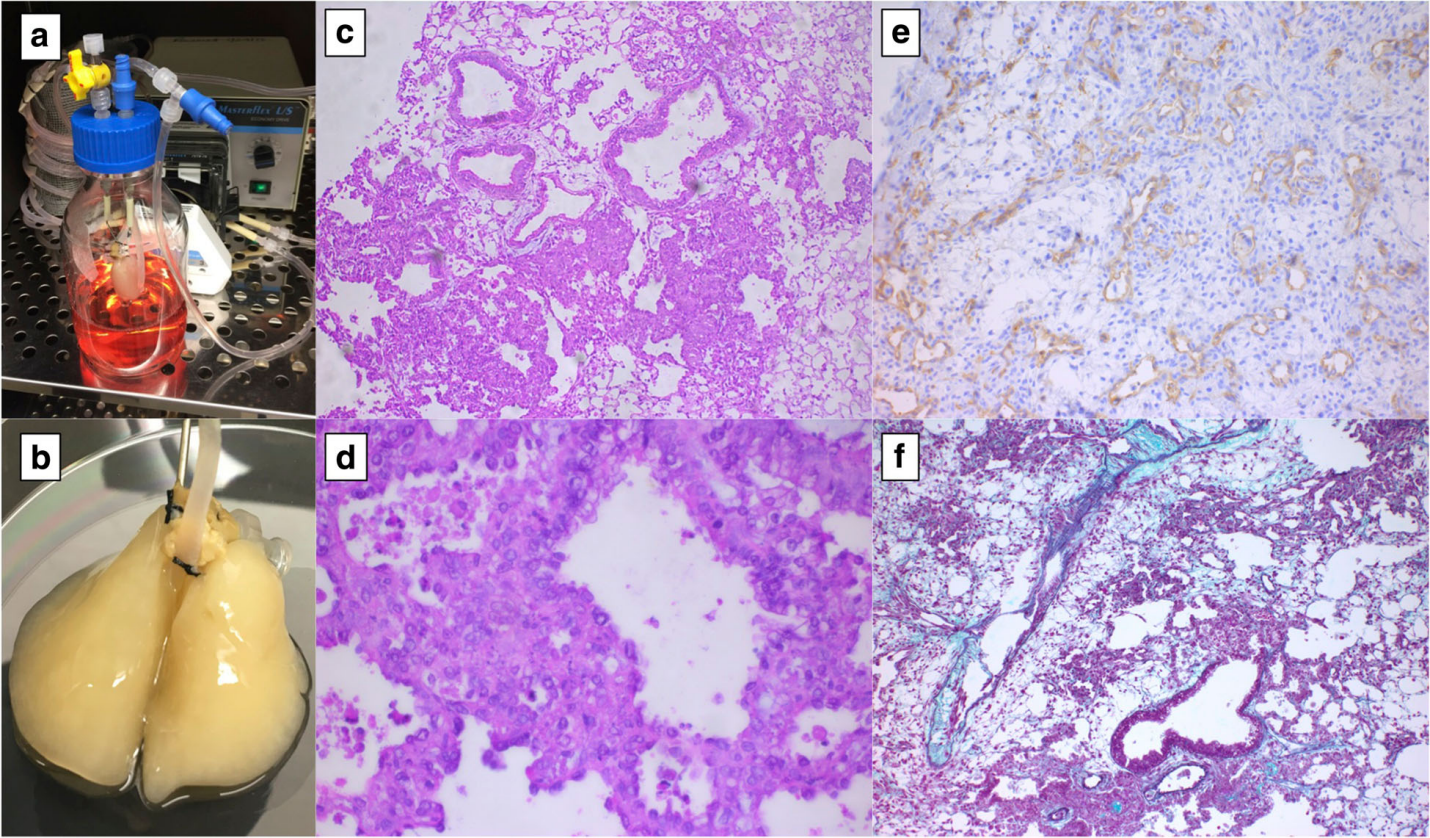
Cellular ex vivo 4D lung model in a bioreactor without any tumor cells (control). Bioreactor showing connection from the lung to oxygenator and pump in cell culture incubator (**a**). The lung after 15 days in the bioreactor is intact (**b**). H&E staining shows intact alveoli, bronchus and vasculature (**c**) with some apoptotic cells. CD34 staining shows the endothelial lining in intact vasculature (**e**) and Movat pentachrome staining represents the components of extracellular matrix (**f**)

A simplified small closed-system bioreactor was set up in an incubator for the lung cell culture (Fig. 1a). We used a custom-designed 500-mL glass bottle with three holes in the cap fitted with a female luer thread-style panel (Cole-Parmer, USA): one for the pulmonary artery cannula, one for the trachea cannula, and one for the circulation of medium from the bottle. To obtain a controlled flow through the pulmonary artery, the cap was connected to a 3-way stopcock (Smith Medical, Dublin, OH, USA). The bottle was filled with 200 mL of complete medium that was circulated through the oxygenator tubing to prevent air bubbles.

Before seeding the human lung cancer cells into the lung matrix, the trachea was cannulated using an 18-gauge needle, and the scaffold was fixed to the bioreactor bottle in a hanging position. To modify it for the metastasis model, we tied the right main bronchus with a silk tie that was left there for the entire experiment and placed it in the bioreactor. The cells diluted in 50 mL complete media were seeded into the left lung lobes through the tracheal cannula via a sterile syringe fed by gravity. We perfused the scaffold at a flow rate of 6 mL/min. The culture medium in the bottle was changed every day to ensure the nutrients were optimal for cell growth, and the CTC were spun down and counted. We grew the cells on the matrix for 10–15 days. The lung matrix was carefully removed from the bioreactor bottle, maintaining sterile conditions, and a lobectomy performed under a culture hood by tying the anatomic lobe with a 2–0 silk tie and resecting it on different days.

### Impact of cellular lung on tumor growth on 2D

We plated 500,000 GFP labeled of A549, H460 and H1299 cells in 6-well plate, with different lung lobes from cellular and acellular rat lungs in cell culture incubator. After 48 h, cells were trypsinized and GFP positive cells were counted using fluorescence activated cell sorting (FACS) with same gating parameters (HMRI NIR Ariall, USA). All the cells were plated from same stock of cells on same day to avoid any bias with the GFP intensity. We determined the percentage of cells that that GFP+ cells in each sample. We compared the groups using student t-test and used *p* < 0.05 as significant.

### Histology and immunohistochemistry

After lobectomy, the lung tissues were placed in 10% paraformaldehyde and shipped to the Pathology Core Laboratory at The Methodist Hospital Research Institute for further processing. Briefly, the tissues were fixed in 10% formalin overnight, processed, and embedded in paraffin. Hematoxylin and eosin (H&E) staining and immunohistochemistry were performed for human mitochondria to identify human cancer regions. The embedded tissues were cut into 4-μm slides and dewaxed; antigen retrieval was performed with antigen-unmasking solution (H-3300; Vector Laboratories, Burlingame, CA) in a steamer for 20 min. Slides were cooled for 20 min at room temperature, washed in PBS, and stained with H&E, Movat Pentachrome (American MasterTech Scientific, CA, USA), Human Anti-mitochondria (Abcam, MA, USA), CD34 and other markers following the standard protocol [10]. Expert board-certified pathologists examined stained slides, and images were captured using a microscope (EVOS, Fisher Scientific, USA). The metastatic lesions per high power field were determined by averaging the number of human mitochondria positive tumor cells per high power field (40X) of 10 areas.

## Results

### Cellular rat lung model

The cellular ex vivo lung was intact in the bioreactor for up to 15 days (Fig. 1b). The control tissue’s histology shows a marginally intact matrix and lung with some apoptotic and non-viable cells (Fig. 1c and d). The lung tissue showed the lung with bronchovascular bundles, alveolar tissue and overlying pleural tissue. The peribronchiolar tissue areas have collections of histiocytes resembling non-necrotizing granulomas (Fig. 1c and d). The alveoli are degenerating and many non-viable cells are seen along with scattered intra-alveolar macrophages. IHC of human mitochondria showed no positive staining, as there were no human cells seeded. Furthermore, staining the lung with Movat pentachrome highlights the matrix architecture showing the presence of collagen, proteoglycans and elastin (Fig. 1f). The immunohistochemistry of CD34 showed its presence in vessels (Fig. 1e).

### Primary tumor in the cellular model

**Fig. 2:**
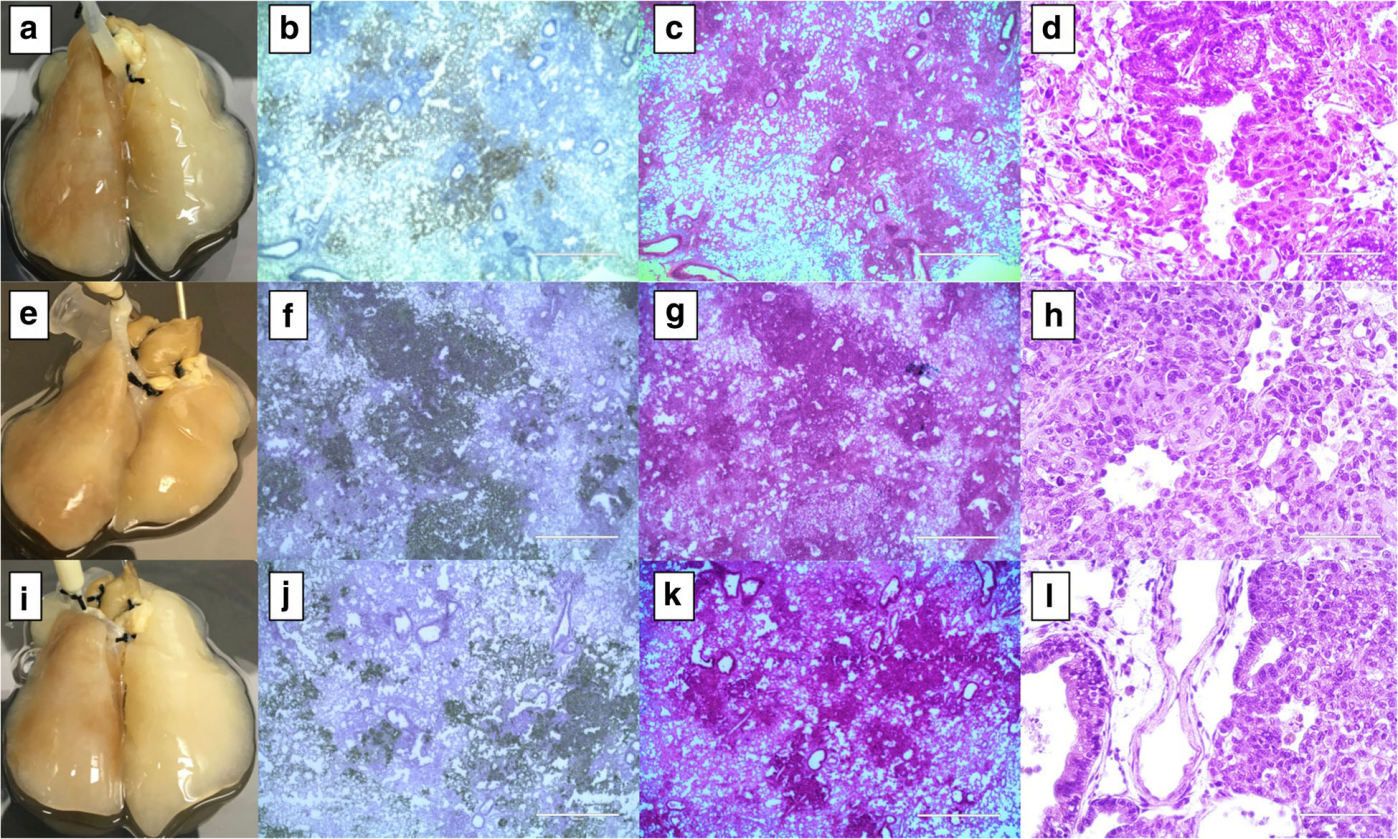
Non small cell lung cancer (NSCLC) cells on the cellular 4D lung model. Primary tumors were formed in a bronchocentric fashion on the cellular lung upon A549 (**a**–**d**), H1299 (**e**–**h**) and H460 (**i**–**l**) cell seeding though the trachea. Human mitochondrial IHC staining clearly shows the presence of a tumor formed by human lung cancer cells in a rat lung after 12 days of culture in a bioreactor. Low and high power H&E staining indicates the presence of a microscopic tumor with distinctive histology based on type of cell placed in the model. A549 cells formed a focal acinar pattern with enlarged nuclei and scattered atypical mitotic cells (**c** and **d**). H1299 cells formed a solid tumor and resemble poorly differentiated cells with scattered atypical mitosis (**g** and **h**). H460 cells formed a solid tumor with vague glandular formation (**k** and **l**)

After 10–15 days of incubation, in the presence of cellular components, human NSCLC, SCLC, and breast cancer cells formed microscopic tumor nodules on a native rat lung though no gross nodules were visualized (Fig. 2a, e and i). These tumor cells showed a distinctive morphology unique to cell type. All of the NSCLC cells (A549, H1299, and H460) formed the solid pattern of a primary tumor in a bronchocentric distribution, but they differ in histology based on subtype. A549, an adenocarcinoma cell line, formed a focal acinar pattern and was compatible with human lung adenocarcinoma (Fig. 2b, c and d). The tumor cells were enlarged epithelioid cells with enlarged nuclei containing prominent nucleoli and scattered atypical mitotic cells (Fig. 2d). The Anti-Human mitochondrial stain highlights the cytoplasmic stain in tumor cells, but not the peribroncholar histocyte aggregates (Fig. 2b). H1299 cell lines also formed primary tumors with a solid growth pattern (Fig. 2f, g and h) and resemble poorly differentiated NSCLC. The cells resembled enlarged epithelioid, large central nuclei with large centrally placed nucleoli. Some of the nuclei are vesicular with scattered atypical mitosis, but this is not a definitive characteristic of squamous or adenocarcinoma (Fig. 2g and h). H460 cell lines also formed tumors on the cellular model with brocho/bronchiolar centric distribution (Fig. 2j, k and l). These tumor cells showed a solid pattern of tumor growth with vague glandular formation and a similar pattern to adenocarcinoma (Fig. 2k and l). Tumor cells appear similar to A549 cells in the cellular model.

**Fig. 3:**
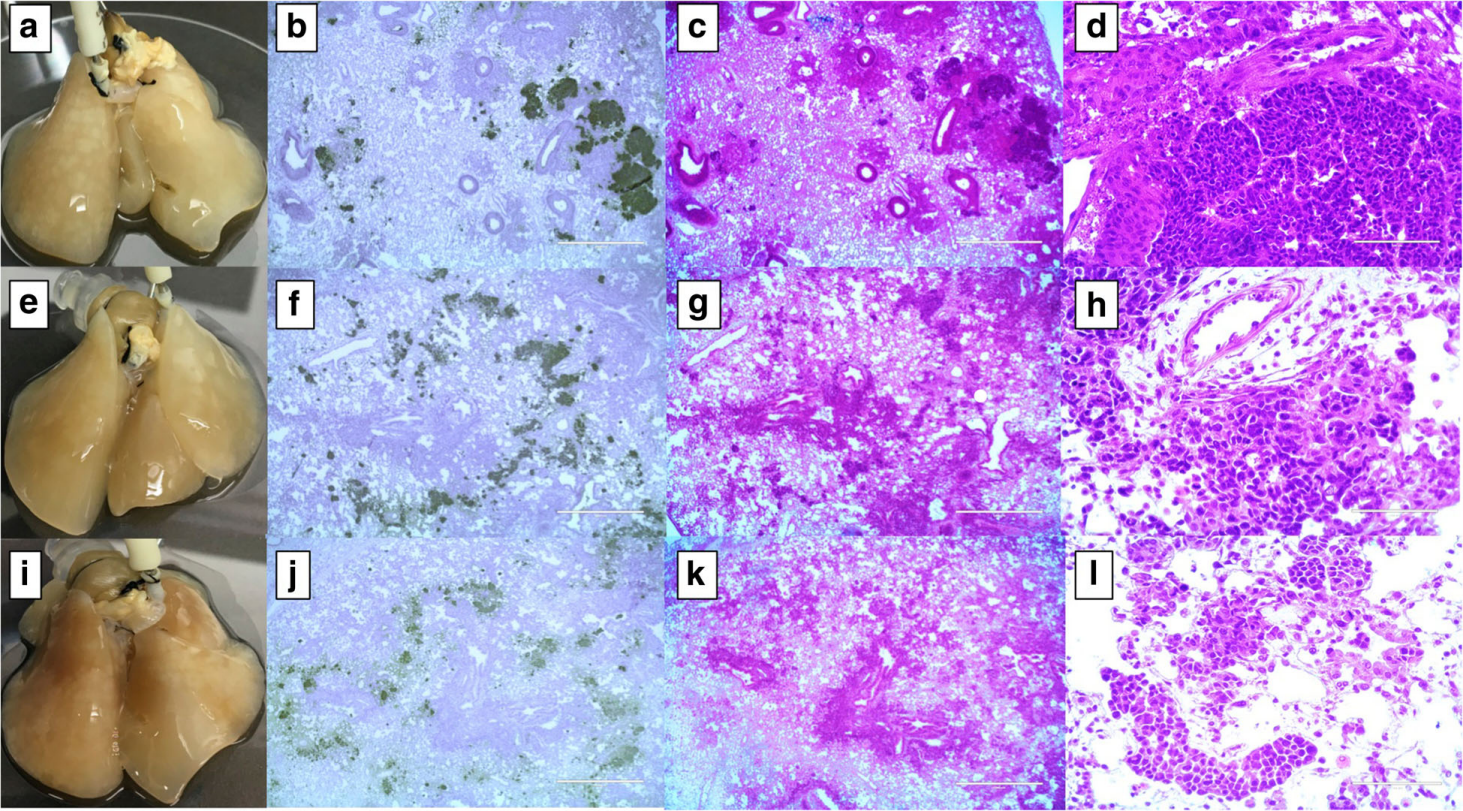
Small cell cancer (SCLC) cells on the cellular 4D lung model. Primary tumors formed in a bronchocentric fashion on the cellular lung upon H69 (**a**–**d**), H446 (**e**–**h**) and SHP77 (**i**–**l**) cell seeding though no distinct nodules appeared. Human mitochondrial IHC staining clearly shows the presence of a tumor formed by human lung cancer cells in a rat lung after 10 days of culture in a bioreactor. Low and high power H&E staining indicates the presence of microscopic tumor with distinctive histology based on the cell type seeded. H69 cells formed tumors similar to high-grade basaloid malignancy with vague rosette/acinar appearance (**c** and **d**). H446 cells formed a collection of tumors, typical of small cell carcinoma (**g** and **h**). SHP77 cells formed a tumor more like a poorly differentiated carcinoma within alveolar spaces (**k** and **l**)

In addition to NSCLC cell lines, we placed small cell lung carcinoma (SCLC) cell lines (H69, H446, SHP77) on the 4D cellular lung model (Fig. 3a, e and i). H69 cell lines showed the primary tumor in the broncho/bronchiolar centric space (Fig. 3b, c and d) with a peculiar pattern of high-grade basaloid malignancy, relatively scant cytoplasm, dark-hyperchromatic nucei, lack of nucleoli, abundant mitosis, and karyorrhectic debris in the background. The tumor was arranged in a nest and there was a vague rosette/acinar appearance (Fig. 3c and d). H446 cells grew on the cellular model and formed a collection of tumors in a bronchiolar centric distribution and intra-alveolar (Fig. 3f, g and h). Cytomorphologic features were typical of small cell carcinoma similar to H69 features. SHP77 cells also formed the primary tumor on the cellular model and appeared more like poorly differentiated carcinoma within alveolar spaces (Fig. 3j, k and l). The tumor cells have large nuclei with some cells having nucleoli. Numerous mitotic cells with associated karyorrhetic debris were also visible (Fig. 3k and l).

**Fig. 4:**
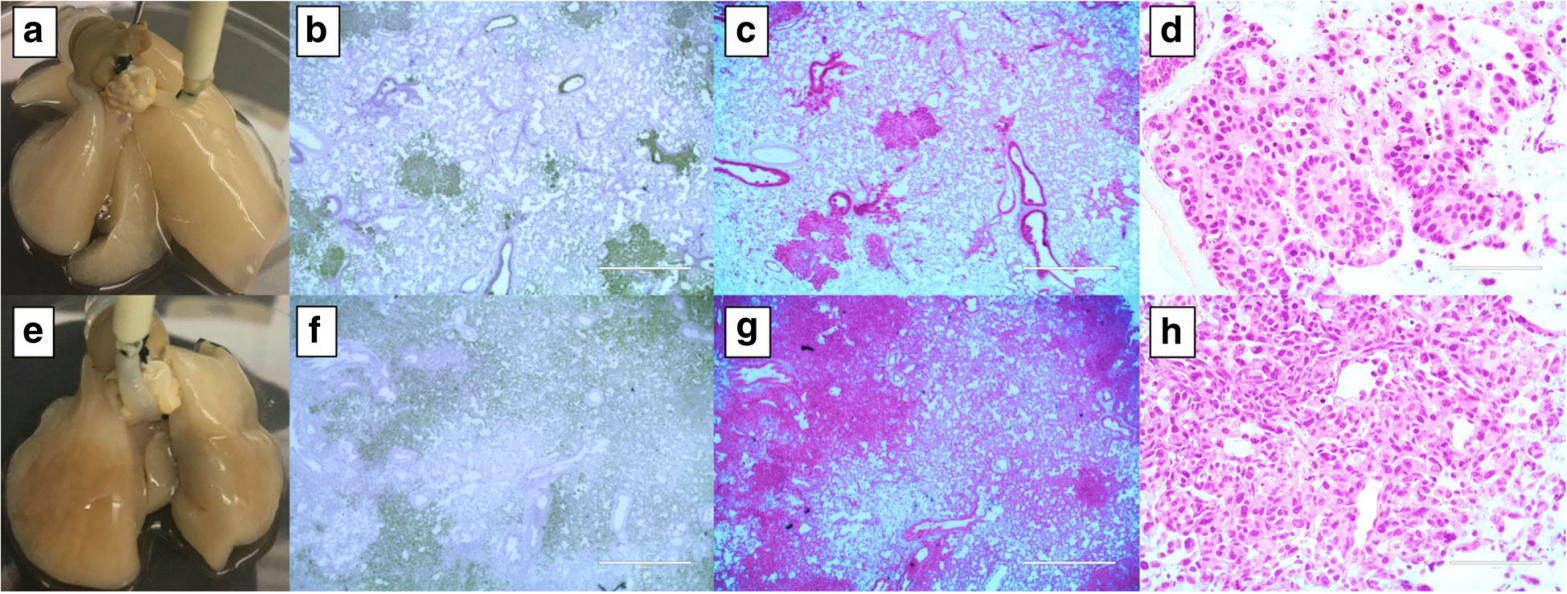
Breast cancer cell lines on the cellular 4D lung model. Primary tumors were formed in a bronchocentric fashion on cellular lung upon MCF7 (**a**) and MDAMB231 (**e**) cell seeding though no distinct nodules appeared. Human mitochondrial IHC staining clearly shows the presence of a tumor formed by human breast cancer cells in a rat lung after 15 days of culture in a bioreactor. Low and high power H&E staining indicates the presence of a microscopic tumor with distinctive histology based of the cell type seeded. MCF7 cells formed solid gland carcinoma with large epithelioid cells and prominent nucleoli (**c** and **d**). MDAM231 cells formed large confluent primary tumors with poorly differentiated malignant carcinoma having spindle cell morphology (**g** and **h**)

Breast cancer cell lines (MCF7 and MDAMB231) also colonized on the cellular 4D lung model and formed microscopic tumor nodules (Fig. 4a and e). An ER/PR positive MCF7 cell formed a primary tumor in the left lobes that resembled a solid gland carcinoma in a broncho-bronchiolar centric distribution (Fig. 4b, c and d). The tumor cells forming the gland were medium to large epithelioid cells with large nuclei, some vesicular and some with prominent nucleoli (Fig. 4c and d). The morphologic features were similar to metastatic high-grade breast carcinoma (ductal). MDAMB231 cells grown on the cellular model formed large confluent primary tumors with poorly differentiated malignant carcinoma (Fig. 4f, g and h). There were loosely cohesive cells with rare acinar formation. Most of the malignant cells have spindle cell morphology with atypical mitosis, looking like metaplastic carcinoma (Fig. 4g and h).

### Circulating tumor cells

**Fig. 5:**
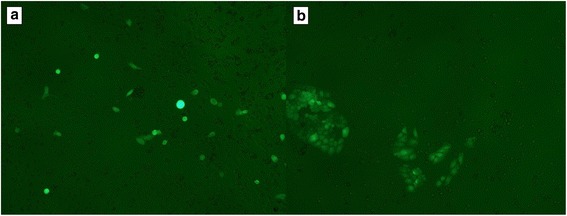
Circulatory tumor cells. We seeded GFP labeled H1299 or H460 tumor cells on the cellular 4D lung model, collected CTC and plated in 96 well plates. Fluorescent microscopy shows CTC from H1299 (**a**) or H460 (**b**) attached to the plate and actively undergoing mitosis

We seeded the cellular model with GFP tagged tumor cells (H1299 and H460). We found GFP positive cells in the circulation and plated them in 96 well plates. These GFP tagged circulatory cells floated and survived in the media for a longer time compared to respective 2D cells. We visualized the viable GFP tagged cells attached to the surface and dividing, under a fluorescent microscope after 7 days (Fig. 5i and j).

### Metastasis to contralateral lung

**Fig. 6:**
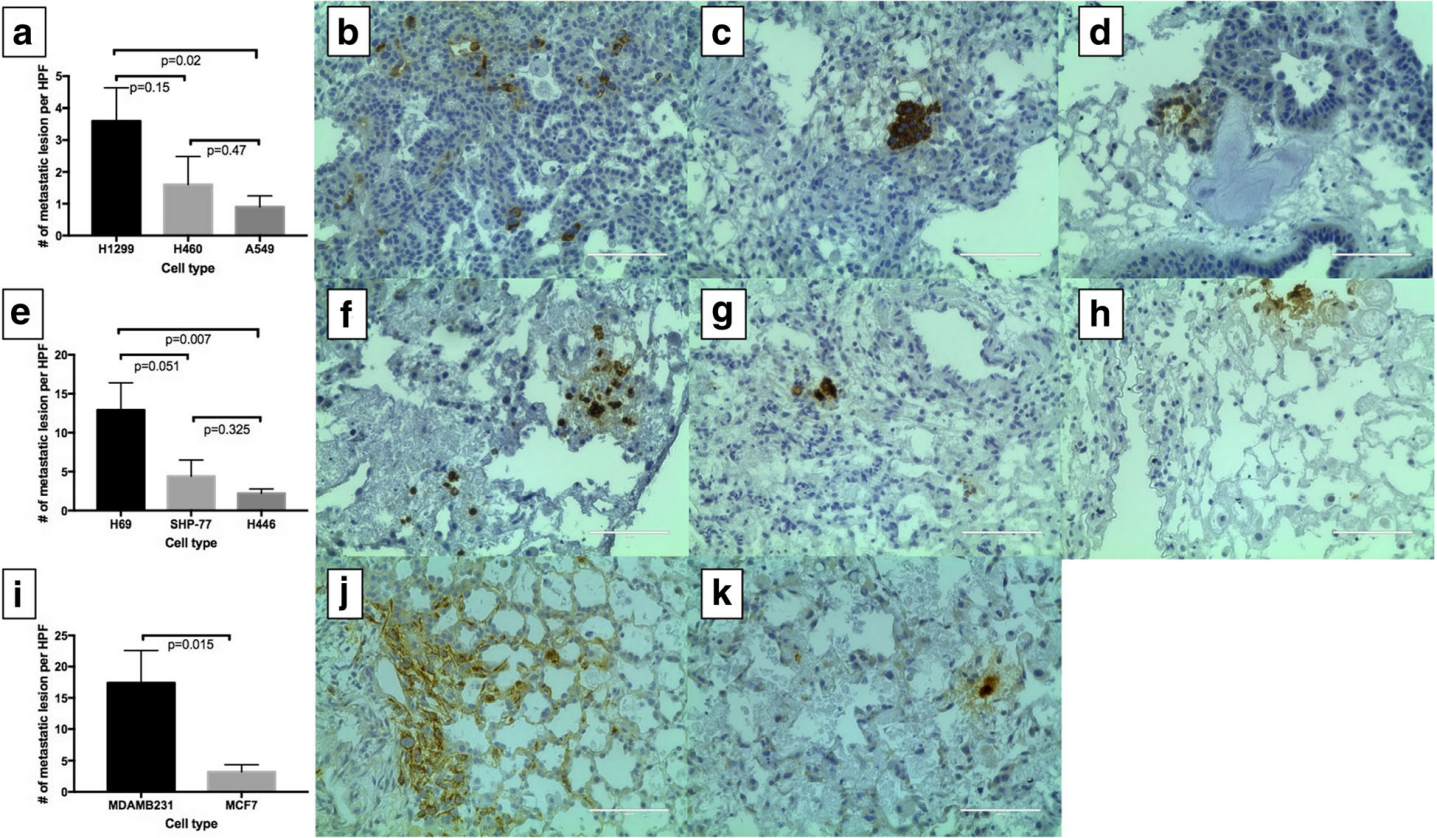
Metastatic lesion formation. Among NSCLC, H1299, a metastatic p53 mutant cell formed significantly more metastatic lesions as compared to A549 cells (**a**; **b** vs **d**) per high power field (HPF). H460 cells also formed metastatic lesions (**a** and **c**). Among SCLC cell lines, H69 formed more metastatic lesions than H446 and SHP-77 (**e**–**h**). In breast cancer cells, MDAMB231, a triple negative cell line, formed significantly more metastatic lesions than MCF-7 cells (**i**–**k**)

The NSCLC, SCLC and breast cancer cell lines formed metastatic lesions that were positive for human mitochondrial cells in the contralateral lung in a vascular distribution (Fig. 6). Unlike the primary tumor, where most of the tumors grew in bronchial distribution, the metastatic lesions were uniformly distributed in the lung. However, there were differences in the number of cells per HPF based on the cell type. Among NSCLC cells, A549 formed the least amount of metastatic lesions (0.9 ± 0.35 cells per HPF), while H1299, a metastatic cell line, formed the maximum number of metastatic lesions (3.6 ± 1.03 cells per HPF). H460 cells showed 1.6 ± 0.88 cells per HPF (Fig. 6a–d). Among SCLC cell lines, H69 formed the highest number of metastatic lesions (12.9 ± 3.49 cells per HPF) as compared to SHP-77 and H446 (4.4 ± 2.09 cells per HPF and 2.2 ± 0.57 cells per HPF) (Fig. 6e–h). MDAMB231, a metastatic breast cancer cell line, formed more metastatic lesions than MCF7 cells (3.2 ± 1.15 cells per HPF vs. 17.4 ± 5.18 cells per HPF) (Fig. 6i–k).

### Impact of cellular lung on tumor growth

**Fig. 7:**
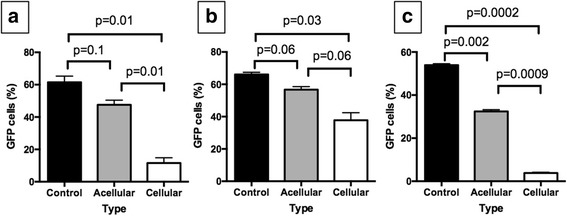
Impact of cellular matrix on tumor growth. We plated GFP labeled H1299 (**a**), H460 (**b**) and A549 (**c**) on a petri dish alone (control) or with acellular matrix (acellular) or cellular matrix (cellular). There was significant decrease in tumor growth with cellular matrix compared to control for all three cell lines

Cellular lung significantly decreased cell growth on 2D of H1299 (*p* = 0.01, Fig. 7a), H460 (*p* = 0.03, Fig. 7b) and A549 (*p* = 0.0002, Fig. 7c) compared to control. In addition, there was significantly lower number of tumor cells in the presence of cellular lung compared to acellular lung for H1299 (*p* = 0.01) and A549 (*p* = 0.0009). There were significantly less A549 cells with acellular lung compared to control (*p* = 0.002).

## Discussion

Tumor models play an important role in understanding tumor progression, metastasis, and cancer therapeutics. To date, there are several 2D/3D in vitro, in vivo and ex vivo cancer models in use, but each one has considerable shortcomings to mimic the human cancer progression [11, 12]. We previously developed an acellular ex vivo 4D lung model for cancer metastasis [2]. The model’s main criticism was that it lacked the normal lung tissue component, and thus the metastasis seen in the acellular ex vivo 4D model could be an artifact of not having cellular components. Thus, we pursued the creation of the cellular model (Additional file 1). We discovered that like the acellular model, the cellular 4D model can mimic tumor progression in 2 weeks and allows for the isolation of a high number of CTC that are the main driver for metastasis. In this study, we successfully grew both NSCLC and SCLC as well as breast cancer cells in the model with all of them forming metastatic lesions.

The cellular lung matrix model is a complex system that supports the growth of human tumor cells in a natural lung microenvironment. The cellular 4D lung model could provide a better model for cancer studies than existing 3D/2D and acellular lung models due to improved cell-cell interactions, cell-ECM interactions and presence of cell populations and a structure that resembles the in vivo environment. Similar to the acellular 4D lung model, the control native lung (devoid of tumor cells) maintains its matrix architecture and leads to tumor development with nutrient perfusion through the vasculature. The major difference lies in the presence of lung cellular components, which the acellular 4D lung model lacks. In both the acellular and cellular model, the tumor grows in an airway centric fashion due to seeding tumor cells through the trachea. Most of the NSCLC tumor cells showed similar histology and tumor cell morphology in the acellular and cellular lung model. The main difference between the cellular and acellular model was the primary tumor volume. We often visualized gross tumor nodules in the acellular model after 2 days while they were not readily visible on the cellular model. Furthermore, on pathologic examination, the nodules were much smaller in the cellular model than the acellular model. We also observed this difference when tumor cells were co-cultured with cellular lung on a petri dish (2D). Several possibilities might account for this difference. One issue is the limited nutrients for the cancer cells in the cellular model. We placed a similar amount of complete media in the cellular and acellular model but the cellular model has a normal cellular component that leaves fewer nutrients for the primary tumor cells. Another possibility is that the normal lung environment may play a tumor suppressive role. We have shown that normal lung fibroblasts can inhibit tumor progression [10]. Thus, it is possible that cancer coordinates with intercellular interactions that are present in normal tissues and disrupt the normalizing cues from the microenvironment, and in turn, the microenvironment evolves to accommodate the growing tumor [13–15].

Compared to the isolation of CTC from in vivo models [16], the process of isolating the CTC is simpler with the ability to tag tumor cells with GFP and sort them with FACS analysis. The GFP-tagged CTC were viable when they were placed on a petri dish similar to the CTC isolated from the acellular model. Moreover, like cells isolated in the acellular model, the CTC were in the supernatant for several days prior to attaching to the petri dish and showed a different phenotype than the parental cells. This feature supports the concept that these cells are a unique phase of tumor development.

Among the NSCLC, the presence of the cellular component impacts the efficacy of metastatic lesion formation. In the acellular model, despite forming fewer CTC, H460 cells tended to form more metastatic lesions compared to H1299 [2] but in the cellular model, there were more metastasis with H1299 compared to H460. In both models, the A549 cells have the least amount of metastatic lesion formation. This deviating behavior may be due to the difference in kinetics of metastatic tumor formation between the models with the presence of an intact tumor microenvironment.

This model also allows for the rapid growth of small cell lung cancer cell lines to form nodules and eventual metastasis. Most of these SCLC cell lines grow slowly and float (less adherent) in vitro which makes them difficult to study [17]. For most in vitro studies, these cells have to be grown in suspension, which does not mimic the growth in patients with small cell lung cancer. When we placed these cells in our model, they formed a primary tumor, CTC and metastatic lesions within 10–14 days. This rapid growth and tumor progression can provide a much-needed new model in understanding the biology of small cell lung cancer.

Furthermore, we were able to show growth of breast cancer cell lines in this model. Breast cancer is the most common cancer in women and the lung is one of the sites of metastasis. We have successfully grown breast cancer cell lines in the acellular model [18] and now we have been able to grow them in the cellular model. Like the NSCLC and SCLC cell lines, the breast cancer cell lines form a primary tumor and metastatic lesions. The MDAMB231, triple negative cell line showed more metastatic cells in contralateral lung lobes than MCF7 cells. This correlates with the aggressiveness of triple negative breast cancer compared to ER + PR+ breast cancer in patients.

## Conclusion

The cellular lung model is an advanced cancer model that mimics the crucial phases of tumor growth—primary tumor, CTC formation and metastatic lesions—in the presence of normal lung tissue. This supports the concept that the natural matrix scaffold is the only component necessary to mimic metastasis and that cellular components modulate the process of metastasis. This ex vivo model can be used to study the complex mechanism of tumor metastasis.

## Additional file

Additional file 1: Schematic diagram illustrating the major steps in ex vivo 4D Lung model creation. (JPG 70 kb)

## Abbreviations

3D: Three dimensional
4D: Four dimensional
ATCC: American Type Culture Collection
CTC: Circulating tumor cells
Ex vivo: Out of the living
GFP: Green fluorescent protein
H&E: Haematoxylin and eosin
IACUC: Institutional animal care and use committee
IHC: Immunohistochemistry
ITGB4: Integrin subunit beta 4
IU: International unit
mg: Milligram
mL: Milliliter
NSCLC: Non-small cell lung cancer
PBS: Phosphate-buffered saline
SCLC: Small cell lung cancer
