# Supplementary figures and images for: Small cell and non small cell lung cancer form metastasis on cellular 4D lung model
Source: BMC Cancer. 2018 Apr 18;18:441. doi: 10.1186/s12885-018-4358-x (PMC5907356; doi:10.1186/s12885-018-4358-x)

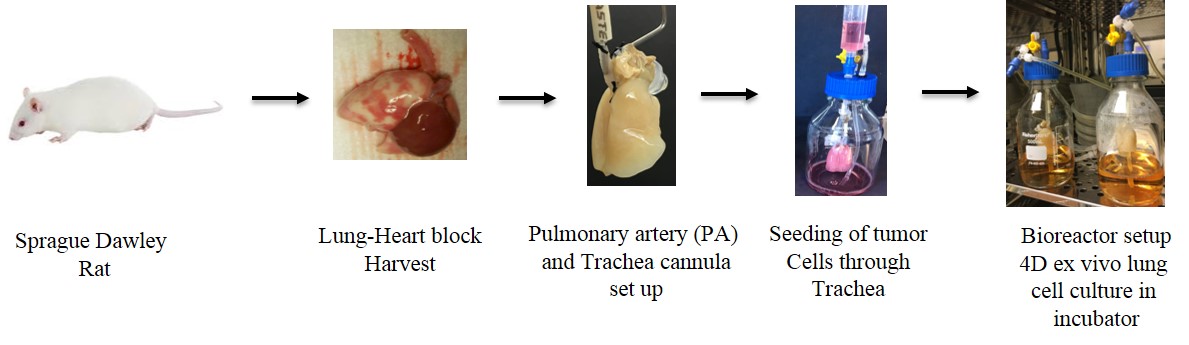

Supplement: Supplementary file 1 — Schematic diagram illustrating the major steps in ex vivo 4D Lung model creation. (JPG 70 kb) [file 12885_2018_4358_MOESM1_ESM.jpg]
